# Systemic Lupus Erythematosus With Multi-System Involvement and Immunosuppression Presenting With a Rare Fungal Brain Abscess

**DOI:** 10.7759/cureus.43575

**Published:** 2023-08-16

**Authors:** Jenna Sutton, Elliot Runge, Ryan Shao, Jill Sharma

**Affiliations:** 1 Internal Medicine, University of Nevada Las Vegas School of Medicine, Las Vegas, USA; 2 Pulmonology and Critical Care, University of Nevada Las Vegas School of Medicine, Las Vegas , USA; 3 Pulmonary and Critical Care Medicine, University of Nevada Las Vegas School of Medicine, Las Vegas, USA

**Keywords:** crainectomy, rheumatology, id critical care, scedosporium apiospermum, disseminated fungal infection, systemic lupus erythromatosus, fungal brain abscess, cns infection

## Abstract

The fungal pathogen *Scedosporium apiospermum* is a ubiquitous opportunistic pathogen found in soil and water that can cause severe infection in hosts with impaired immunity. Patients with systemic autoimmune diseases such as systemic lupus erythematosus (SLE) are already at risk for infections given their altered immunity. This can be exacerbated further in patients taking immune-suppressing medications to control their disease status. Here, we present a case of a rare and challenging clinical scenario of a woman with refractory multi-organ SLE on steroids who developed neurologic deficits found to have a brain abscess caused by a unique fungal etiology.

## Introduction

Systemic lupus erythematosus (SLE) is a chronic autoimmune disease characterized by the production of autoantibodies against self-antigens, resulting in widespread inflammation and multi-organ involvement. This disease can affect almost all organ systems, leading to a vast array of clinical manifestations and complications [1]. Cutaneous manifestations such as the classic malar rash and photosensitivity are common; joint involvement, including arthritis and arthralgia, also occurs in the majority of patients. Other organ systems affected by SLE include the cardiovascular system (pericarditis/myocarditis, nonbacterial thrombotic endocarditis, coronary artery disease), hematologic system (resulting in anemia, leukopenia, and thrombocytopenia), pulmonary system (pleuritis and pulmonary embolism), gastrointestinal system (vasculitis and mesenteric ischemia), and central nervous system (neuropsychiatric lupus) [2].

The management of SLE primarily focuses on controlling disease activity and preventing flares. Glucocorticoids are the mainstay of treatment for acute flares due to their potent anti-inflammatory effects. However, prolonged use of high-dose steroids can lead to immunosuppression, increasing susceptibility to infections. Immunosuppressive and immunomodulatory agents, such as hydroxychloroquine, methotrexate, mycophenolate mofetil, azathioprine, and cyclophosphamide, are commonly used to reduce the steroid burden to achieve disease control [3]. While these medications are effective in controlling disease activity, with prolonged immune suppression these patients are at increased risk for opportunistic infections [4].

Central nervous system (CNS) infections of fungal etiologies are relatively rare but can occur in immunocompromised individuals and can have devastating consequences [4,5]. As presented in this case, the patient had long-standing SLE and was previously hospitalized for a lupus flare treated with greater than four weeks of high-dose steroids. She presented with focal neurologic deficits and was later found to have a brain abscess for which fungal cultures were positive for the organism *Scedosporium apiospermum*, a rare fungal pathogen. There have been prior case reports in the literature of this specific organism causing CNS infections. One such case by Gao et al. presented a clinically similar patient in an immunocompromised state in which a fungal pathogen in the same genus caused a brain abscess [6]. Both voriconazole and itraconazole have been shown to be effective against *Scedosporium spp*. and thus the empiric use of these antifungal agents is the current recommended treatment [6]. We report a case of a patient with refractory SLE previously on long-term steroid use presenting with new neurologic changes found to have a brain abscess caused by the rare fungal pathogen *S. apiospermum* that was diagnosed after craniotomy. 

## Case presentation

A 41-year-old female with a history of SLE was admitted to the hospital with new-onset neurologic deficits. The patient had multiple organ involvement in her SLE which included antiphospholipid syndrome manifesting as prior strokes requiring chronic warfarin therapy, chronic hemolytic anemia, lupus nephritis, and lupus pleuritis. Due to her chronic SLE complications, the patient had previously escalating immunosuppressive therapies including hydroxychloroquine 200 mg BID, azathioprine 50 mg daily, and prednisone 5mg daily. After a recent hospitalization for autoimmune hemolytic anemia and lupus nephritis earlier the same year, her dose of prednisone was increased to 60mg daily. Around two months later, she was again hospitalized with diffuse alveolar hemorrhage manifesting as bilateral ground glass opacities for which her steroid dose was again increased to prednisone 150 mg twice daily after a full negative infectious work-up. The patient was instructed to follow up at an outpatient rheumatology appointment where her medications were again adjusted including the initiation of a prednisone taper. 

**Figure 1:**
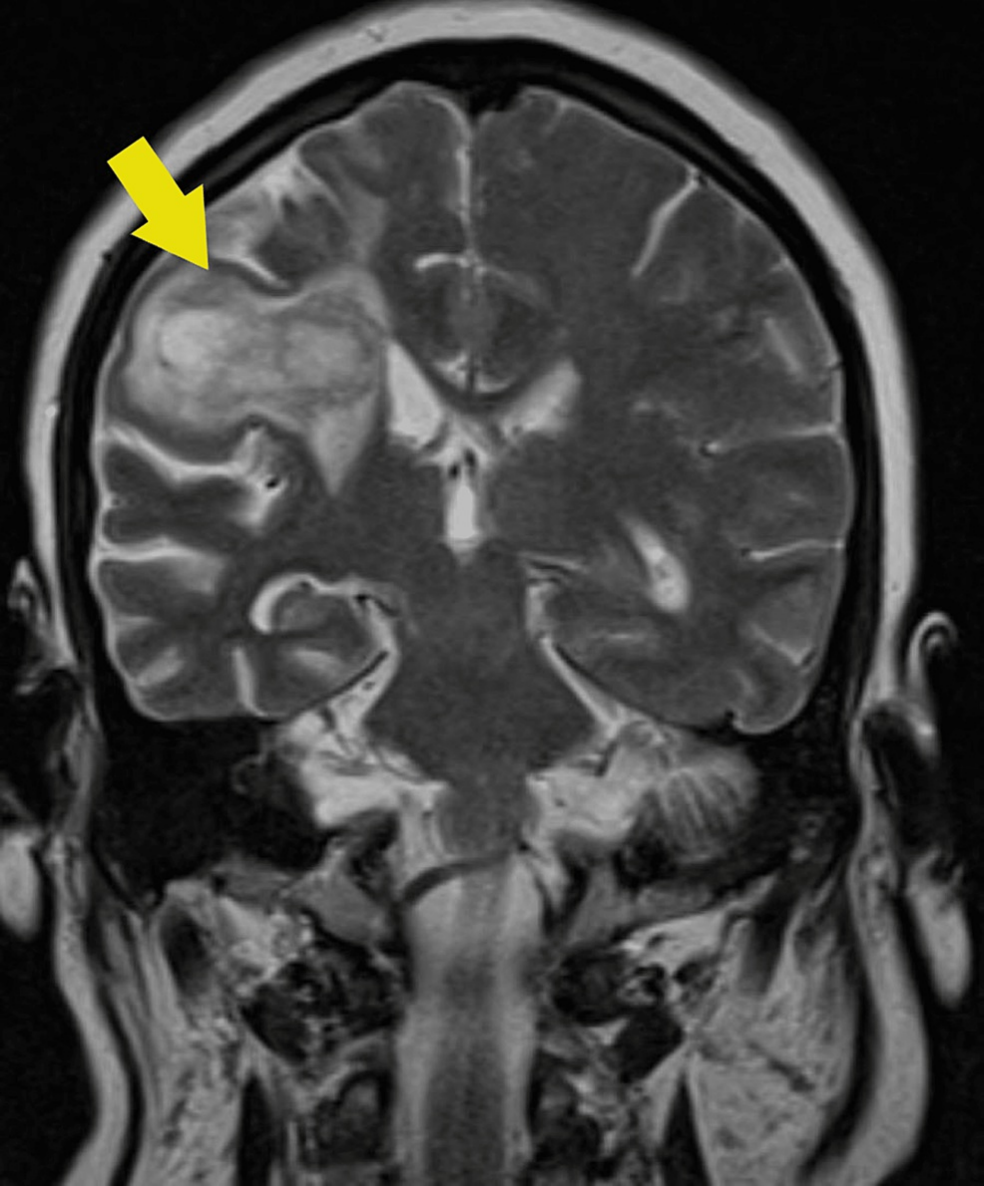
MRI brain coronal view. The yellow arrow indicates the location of the abscess

**Figure 2:**
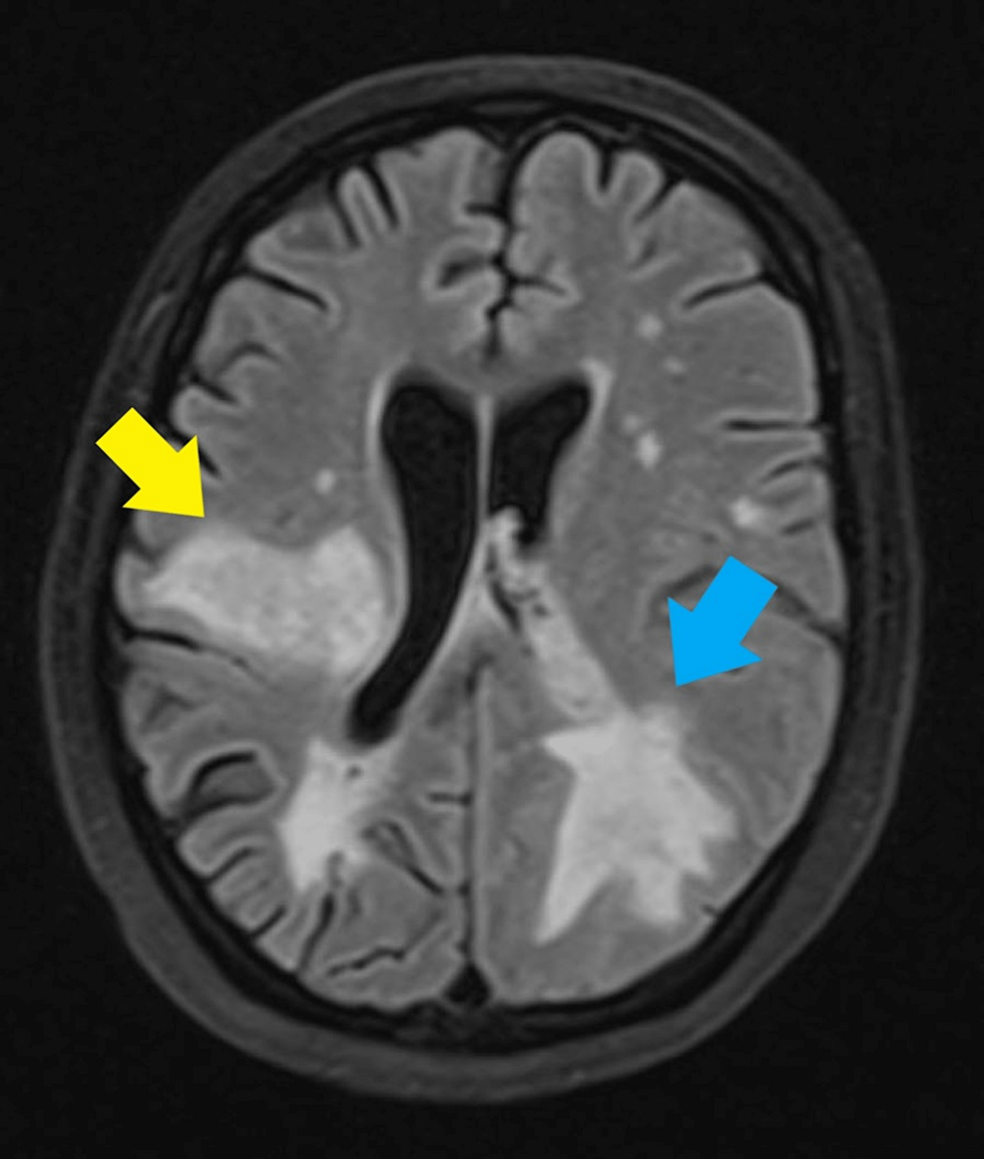
MRI brain axial view The yellow arrow shows the location of the abscess and the blue arrow shows the location of intracerebral hemorrhage with intraventricular hemorrhage

She reportedly was doing well at home until one day prior to presentation when she developed acute onset left-sided weakness. She had been reportedly noncompliant with her medications for several days prior to admission. Upon admission, her home immunosuppressant medications were held with the exception of her prednisone taper which was continued after consultation with rheumatology. The patient was admitted to the medical intensive care unit for one-hour neurological checks and worked up with imaging to include CT head without contrast and MRI brain with and without contrast. Notably, her MRI demonstrated a large left occipital abscess with extension into the left lateral ventricle with vasogenic edema (Figures 1, 2). It also showed a developing 4.6 x 2.4 cm right posterior frontal abscess with multiple small acute to subacute infarcts in the right parietal and occipital lobes. Neurosurgery was immediately consulted, and the patient underwent a left occipital craniotomy for evacuation of the abscess with the placement of an external ventriculostomy drain (EVD). Intraoperatively, frank pus was noticed upon excision of the abscess in the left occipital region and intraoperative cultures were obtained. She had no intraoperative or immediate postoperative complications from the surgery and was continued on broad-spectrum antibiotics and antifungal agents including amphotericin, linezolid, cefepime, and metronidazole per infectious disease team recommendations. The prednisone dose was continued during this stage of her hospitalization. On postoperative day 1, she underwent a repeat CT head which demonstrated pneumocephalus and hemorrhage in the left frontal horn which was suspected to be causing increased intracranial pressure. Therefore, the patient was taken back to the operating room for placement of a left transparietal ventriculostomy shunt. The patient was extubated after the surgery and remained stable in the ICU during postoperative days 2-4 with specific EVD, blood pressure, and sodium monitoring. 

On postoperative day 2 of her clinical course, the differential for infectious etiologies remained broad and as such, the decision was made to add intravenous voriconazole to the patient's medication regimen after intraoperative cultures demonstrated filamentous fungal elements. Over the next couple of days, her clinical course was largely uncomplicated. However, it was noted that she did not have any significant improvement in her neurological status as compared with her presenting symptoms. On postoperative day 8, fungal cultures returned positive for *Scedosporium apiospermum*. Since the sensitivity of the organism to various antifungal agents was not back yet at this point, the infectious disease team recommended continuation of concomitant liposomal amphotericin B and voriconazole. Due to her absence in expected neurologic exam improvement, the neurosurgery service recommended a repeat brain MRI to evaluate for EVD and intraventricular shunt placement. This repeat MRI demonstrated enlargement of the patient’s bilateral brain abscesses as well as progression of the right-sided acute to subacute infarcts. Due to the patient's persistent left hemiparesis, neurosurgery made the decision to take the patient back to the OR for a right-sided craniotomy and abscess evacuation. Intraoperatively, there was frank pus again in the right posterior frontal abscess; cultures were taken. The prior EVD was left in place. The patient remained clinically stable following her second operation and was eventually able to have her care transitioned to the hospitalist team.

Her neurologic deficits persisted, but the patient otherwise had an uneventful course on the medical floor. However, she started to develop worsening kidney function indicative of acute kidney injury and decreasing urine output despite holding all nephrotoxic medications. She developed fevers, diarrhea, increased dyspnea, tachycardia, and increasing leukocytosis. Empiric broad-spectrum antibiotics were started, but she was unfortunately found to be both *Clostridioides difficile* and SARS-CoV-2 positive. She received fidaxomicin, steroids, and remdesivir, but her clinical state worsened considerably including increasingly altered mental status and worsening kidney function ultimately requiring renal replacement therapy. She was transferred back to the ICU where a dialysis catheter was inserted and hemodialysis was initiated. During her ICU course, the patient developed worsening respiratory status including increased work of breathing and decreased oxygenation. Serial chest imaging showed development of bilateral airspace opacities indicative of acute respiratory distress syndrome (ARDS). The patient was intubated and placed on lung protective ventilation. The patient was then placed on increasing doses of vasopressors for hypotension but unfortunately quickly developed evidence of multiple organ failure. Inhaled epoprostenol and proning maneuvers were attempted due to worsening ARDS; however, she continued to decompensate, prompting several in-depth goals of care discussions with the family. Unfortunately, the patient continued to decompensate clinically and the decision was made to withdraw care. The patient expired later that day. 

## Discussion

*Scedosporium* species, including *S. boydii *and *S. apiospermum*, are filamentous fungi found in soil, sewage, and polluted water [7]. The genus represents an opportunistic pathogen that has been recognized for its potential to cause invasive infections in humans [8]. Infections most frequently result from trauma or surgery but may also occur from inhalation of spores from the environment [7,9]. In an immunocompetent host, infection from these organisms are usually localized to soft tissue infections such as mycetoma or osteoarticular infections and are associated with *S. boydii *species [10]. However, in immunosuppressive conditions such as HIV, post-transplant patients, poorly controlled autoimmune diseases, or even diabetes mellitus, the infection can disseminate hematogenously [10]. A case by Montejo et al. describes a fungal brain abscess caused by *S. apiospermum* in a renal transplant recipient patient, one of the first studies to attempt to elucidate the connection between an immunosuppressed state and acquisition of disseminated infection from this species. Through a literature review of existing case reports, they concluded that the degree of immunosuppression is one of the most important factors in the likelihood of infection, and reversal of immunosuppression is essential to survival [11]. Prior to our patient’s final admission, she was admitted five times within a four-month period for SLE complications that resulted in increasing immunosuppressive therapy. 

Upon review of the current literature surrounding *Scedosporium spp. *and its propensity to cause CNS infections, it becomes apparent that clinical signs and symptoms, basic laboratory analysis, and even CSF studies lack adequate specificity to provide a diagnosis [12]. Thus, a rapid and definitive diagnosis and treatment plan should be made based on culture identification, and sensitivities based on molecular next-generation sequencing techniques (mNGS). In our scenario, an intraoperative culture was taken and processed in-house, but a specimen had to be sent out for mNGS testing. A similar case to the one presented here described a more novel approach to diagnosis using matrix-assisted laser desorption ionization-time of flight mass spectrometry to detect *Scedosporium *spp, [12]. While not offered at every hospital, the matrix-assisted laser desorption/ionization-time of flight mass spectrometry system is a spectrometry-based assay that helps differentiate between fungal species for rapid laboratory identification which could decrease the time between patient presentation and receiving appropriate antifungal therapy [12]. This is important because the mortality of *Scedosporium spp* related infections remains between 50 and 70%; however, addition of antifungal therapy can have an impact on clinical course and a reduction in mortality [13-16]. 

Our case illustrates the delicate balance between adequate immunosuppression for refractory autoimmune diseases, such as lupus, and mitigating the risk of opportunistic infections. Providing a swift and accurate diagnosis of infectious etiologies in immunocompromised patients is essential for survival. It is important to note that opportunistic infections can present with a variety of symptoms including CNS, cutaneous, ocular, GI, and pulmonary, and our differential must remain broad. Our patient presented with neurologic deficits, which was concerning for acute stroke, particularly with the overall absence of infectious signs or symptoms. However, due to stat head imaging performed on admission, the CNS abscess was discovered on hospital day 1, triggering prompt neurosurgical intervention. While cultures were obtained from the operating room early in the hospital course, the true identity of the pathogen was not discovered until a week into the hospitalization. Our patient was placed on empiric amphotericin initially until postoperative day 2 when voriconazole was added on after fungal elements were identified from the culture possibly representing a slight delay in appropriate antifungal coverage. 

Current treatment guidelines for CNS infections due to *Scedosporium *species include surgical drainage combined with voriconazole [13]. However, mortality rates remain high (50-70%), particularly for immunocompromised patients [14-16]. Despite rapid identification of our patient’s cerebral abscess and the inciting pathogen, followed by rapid initiation of appropriate therapy, our patient did not survive. Ultimately, the patient's decompensation toward the end of her clinical course occurred from a multitude of secondary infections including C. difficile colitis and severe COVID pneumonia resulting in acute respiratory distress syndrome. Prompt diagnosis of infectious organisms in immunocompromised patients is always of utmost importance; however, mortality for invasive fungal CNS infections remains high even with early detection. The patient had presented to the hospital with a high home prednisone dose which may have been the precipitating factor in this case highlighting the importance of appropriate treatment with immunosuppression only to the extent that is necessary to prevent such devastating complications. 

## Conclusions

*S. apiospermum* is a ubiquitous opportunistic pathogen found particularly in soil and water that has been shown to cause severe infections in hosts with impaired immunity. Patients with systemic autoimmune diseases such as SLE are already at risk for opportunistic infections due to their altered immunity. In summary, this case and its findings underscore the importance of prompt diagnosis with novel techniques of uncommon infectious causes in patients with chronic autoimmune conditions, the consideration of the immune status of the patient, and the early initiation of treatment for management of not only CNS *Scedosporium* infections but for appropriate treatment with immunosuppression only to the extent that is needed. Further research is needed to facilitate rapid diagnosis of invasive fungal pathogens and refine treatment guidelines with the goal of improving mortality outcomes for patients who present with rare, opportunistic infections in the setting of immunodeficiency. 
